# Effects of Sublethal Cadmium Exposure on Antipredator Behavioural and Antitoxic Responses in the Invasive Amphipod *Dikerogammarus villosus*

**DOI:** 10.1371/journal.pone.0042435

**Published:** 2012-08-03

**Authors:** Pascal Sornom, Eric Gismondi, Céline Vellinger, Simon Devin, Jean-François Férard, Jean-Nicolas Beisel

**Affiliations:** Laboratoire des Interactions, Ecotoxicologie, Biodiversité, Ecosystèmes, CNRS UMR 7146, Université de Lorraine, Metz, France; Hokkaido University, Japan

## Abstract

Amphipods are recognised as an important component of freshwater ecosystems and are frequently used as an ecotoxicological test species. Despite this double interest, there is still a lack of information concerning toxic impacts on ecologically relevant behaviours. The present study investigated the influence of cadmium (Cd), a non-essential heavy metal, on both antipredator behaviours and antitoxic responses in the invasive amphipod *Dikerogammarus villosus* under laboratory conditions. Amphipod behaviour (i.e. refuge use, aggregation with conspecifics, exploration and mobility) was recorded following a 4-min test-exposure to 500 µg Cd/L with or without a 24-h Cd pre-exposure and in the presence or absence of a high perceived risk of predation (i.e. water scented by fish predators and injured conspecifics). Following behavioural tests, malondialdehyde (MDA) levels, a biomarker for toxic effect, and energy reserves (i.e. lipid and glycogen contents) were assessed. Cd exposures induced (1) cell damage reflected by high MDA levels, (2) erratic behaviour quantified by decreasing refuge use and exploration, and increasing mobility, and (3) a depletion in energy reserves. No significant differences were observed between 4-min test-exposed and 24-h pre-exposed individuals. Gammarids exposed to Cd had a disturbed perception of the alarm stimuli, reflected by increased time spent outside of refuges and higher mobility compared to gammarids exposed to unpolluted water. Our results suggest that Cd exposure rapidly disrupts the normal behavioural responses of gammarids to alarm substances and alters predator-avoidance strategies, which could have potential impacts on aquatic communities.

## Introduction

Aquatic ecosystems are constantly being exposed to chemical contaminants from industrial, agricultural and domestic sources. In recent decades, metals with no significant biological function, such as cadmium (Cd), have received particular attention due to their high ecotoxicity, even at very low concentrations, and their ability to bioaccumulate in many aquatic species [1]. Cd is a heavy metal toxicant that occurs naturally in the environment, in insignificant amounts; however, its impact is steadily increasing due to anthropogenic activities. Freshwater crustaceans are amongst the most sensitive of macroinvertebrate species to Cd [2]. This is especially so for gammarids, which are increasingly used as biological models in ecotoxicological studies. In addition to its ability to bioaccumulate and its adverse effects on survival, Cd has been shown to significantly affect an organism’s behavioural patterns, including feeding, ventilation and locomotion [3]–[5]. Cd is also known to affect the transfer of chemical information between organisms [6]. Indeed, the phenomenon of Cd-induced info-disruption has been shown to impact on anti-predator behaviour in many aquatic species, including fish and crustaceans [7], [8]. Although several studies have been devoted to the effects of heavy metals such as Cd on gammarids, the species most often used are either native or naturalised. Very little information is available on the responses of invasive European amphipods to chemical stress, despite a number of species now reaching dominant levels in some European waters.

In recent decades, a number of exotic amphipod species have increased their ranges in Europe, spreading west from their native Ponto-Caspian region. One of these, *Dikerogammarus villosus*, has become well established in the River Moselle (north-eastern France) since its first appearance in 1999 [9]. Its invasive success has been helped by life-history traits, predatory behaviour and reproductive characteristics [10], [11] that have resulted in *D. villosus* becoming one of the dominant freshwater amphipod species in many large European hydrosystems. Due to its recent wide distribution and high densities in European inland waters [12], *D. villosus* is rapidly becoming a classical model species used in ecotoxicological tests to develop biomarkers [13] or assess effects of pollutants [14], [15].

Many tools have been developed in amphipod testing to estimate and predict the effects of contaminants on organisms, the most widely used ecotoxicological endpoints being survival, growth, food consumption and assimilation, moult frequency, reproduction, enzymatic biomarkers and osmoregulation. The assessment of sublethal ecotoxicity is of ecological relevance as mortality does not always occur in organisms exposed to pollutants. In such cases, behavioural changes are relevant tools for ecotoxicity testing and water quality monitoring [16]. Indeed, behavioural endpoints, previously described as “early warning responses” to toxicants and environmental stresses [17], are sensitive, fast and relatively easy to assess, and are cheap, non-invasive and useful indicators of sublethal exposure in both laboratory and field conditions. They are highly ecologically relevant and they have the potential to link physiological functions to ecological processes, e.g. locomotion is required not only to find food, to obtain mates and to migrate, but also to escape predation.

Amphipods constitute the prey of various upper trophic-level predators including other invertebrates, vertebrates and especially fish [18]. Hence, in the lower River Rhine, *D. villosus* rapidly became a regular food item for eel [19], while in Upper Lake Constance, five years after its first observation, *D. villosus* had readily been included into the diet of zoobenthivorous fish [20]. In aquatic environments, prey are able to assess the presence, activity and hunger of predators [21] through chemical signals [22], in addition to via visual, hydrodynamic or auditory cues [23], [24]. Chemical cues can include the scent of the predator itself [25], that of its prey [26], or the scent emitted by injured conspecifics [27], [28]. Earlier studies devoted to antipredator mechanisms in amphipods have highlighted their sensitivity to both fish scent [29] and injured conspecifics [30], [31]. In order to avoid encounters with predators, amphipod prey can display chemical, structural or behavioural defences [32] such as increased refuge use and decreased locomotion activity, which have been shown to increase survival [30]. Further, gregarious behaviour, a form of cover-seeking, tends to decrease individual risk of predation in various organisms, including amphipods [33].

The aims of the present study were (1) to investigate antipredator responses of *D. villosus* to a predation alarm cue and (2) to test the hypothesis that Cd may interfere with the perception of predation risk chemical signals. In laboratory experiments, the perception of predation risk will be determined by measuring behavioural biomarkers such as refuge use, aggregation with conspecifics, environmental exploration and mobility. In parallel, we will measure malondialdehyde (MDA), a lipoperoxidation product used as a biomarker of toxic effect, and lipid and glycogen levels, to monitor energy expenditure in the organism. As gender has previously been shown to be a confounding factor in physiological biomarker responses assessment [34], [35], all measurements were carried out separately on males and females.

## Materials and Methods

### Ethics Statement

All experiments therein have been conducted in accordance with current laws in France, including the European Convention for the Protection of Vertebrate Animals used for Experimental and Other Scientific Purposes, Strasbourg, 18.III.1986 (Annex B). All organisms unexposed to toxicants (i.e. unused and untested amphipods and all fish) were later released at their respective sampling sites within one week. The sampling site locations are not privately-owned or protected in any way and macroinvertebrate sampling at these locations did not require any specific permit. The field studies did not involve endangered or protected species. The bullhead *Cottus gobio* is listed in Annex 2 of Council Directive 92/43/EEC of 21 May 1992 on the Conservation of Natural Habitats and of Wild Fauna and Flora. In France, however, *C. gobio* is not a protected species and its use in scientific research does not require any specific permit. Fish sampling was performed by the Office National de l’Eau et des Milieux Aquatiques (ONEMA), the French national agency for water and aquatic environments, a state-owned company licensed to conduct scientific fishing, allowing sampling and the transport of the animals to the laboratory. All experiments were supervised by the holder of a Level 1 authorisation to conduct experiments on animals, as approved by the French Ministry of Agriculture and Fisheries (Dr. Michael Danger).

### Sampling and Housing

Specimens of *D. villosus* were collected using pond nets (5 mm mesh) during spring 2011, from an unpolluted station on the River Moselle in north-eastern France (Saulcy; 49°07′N and 6°10′E). We selected intermediate-sized adults of around15 mm in order to avoid potential bias induced by gammarid length/age. Similarly, ovigerous females were not sampled in order to standardise physiological condition. Specimens were brought to the laboratory in 3 L opaque plastic tanks filled with water taken on site within one hour of sampling. Leaves collected from the site were placed into each tank in order to reduce the stress induced by handling and transport. In the laboratory, specimens were maintained at a maximum density of 20 individuals/L in plastic aquaria (31 cm long, 28 cm wide, 14 cm high) filled with dechlorinated, UV-treated, and oxygenated tap water. Glass pebbles (15 mm diameter, convex side down) and artificial plants were added to provide refuges and minimise cannibalism. Gammarids were fed *ad libitum* with alder leaf disks and chironomids. The room’s temperature was kept constant at 18±1°C and the photoperiod kept at 12 h light:12 h dark. Amphipods were housed under these conditions for 72 h before pre-exposures.

The bullhead *C. gobio* was chosen as the test predator as it is a native and widespread fish species in north-eastern France. This typical benthic-feeding fish is known to include amphipods in its diet and is frequently used to assess invertebrate antipredator behavioural responses [36], [37]. Twenty fish of similar size (approx. 9 cm) were collected from the River Moselle in spring 2011 using a pond net (5 mm mesh). Bullheads were transported to the laboratory in a 30 L aerated opaque plastic tank filled with water collected on site within one hour of sampling. In the laboratory, specimens were maintained at a density that did not exceed one fish/L in aquaria (60×29×31 cm) filled with 6 L of dechlorinated, UV-treated and oxygenated tap water. To each housing unit, we added coarse gravel and stones (>1 cm diameter) and some artificial plants to minimise aggression between individuals. The aquaria were maintained under the same temperature and photoperiod conditions as those for amphipods and the fish were maintained under these conditions for one week prior to the start of experimentation. During this time, the fish were fed *ad libitum* with living *D. villosus* taken randomly from the sample site. Bullheads were deprived of food for 72 h prior to use for preparation of scented water.

### Scented Water

Scented water was prepared using a protocol similar to that described in [38]. Six previously starved bullheads were placed in a plastic aquarium (31×28×14 cm) filled with 6 L of dechlorinated, UV-treated and oxygenated tap water for 24 h. Thirty amphipods were offered to the bullheads in order to obtain a mean of five predation events/fish/L over 24 h. The procedure was repeated for each test condition and was expected to provide a predation signal close to that observed under natural conditions, which includes both predator odour and chemical cues released by injured amphipods [31], [39]. Behavioural tests were performed within 24 h following the preparation of scented water.

### Cadmium Solutions

A toxic stock solution was prepared by dissolving cadmium chloride (CdCl_2_) in dechlorinated and UV-treated tap water. The stock solution was stabilised with 0.1% of HNO_3_ (65%, Suprapure, Merck). Cd-spiked test solutions were prepared through dilution of the stock solution in either unscented or previously prepared scented water in order to obtain a nominal concentration of 500 µg Cd/L.

### Pre-exposure and Test Exposure Conditions

For all experiments, temperature and photoperiod were identical to those for amphipod housing. During pre-exposures, 120 specimens of *D. villosus* were placed in plastic aquaria (31×28×14 cm) filled with 6 L of oxygenated test solution, where they were kept unfed for 24-h prior to the behavioural tests. Amphipods were exposed to three conditions according to pre-exposure and test exposure: (1) Control - 24-h pre-exposure to Cd-free water (i.e. dechlorinated and UV-treated tap water) and 4-min test exposure to Cd-free water; (2) Cd-free pre-exposure - 24-h pre-exposure to Cd-free water and 4-min test exposure to 500 µg Cd/L; and (3) Cd pre-exposure - 24-h pre-exposure to 500 µg Cd/L and 4-min test exposure to 500 µg Cd/L. These conditions were replicated twice, both with and without the addition of chemical signals from the predator and injured conspecifics in test solution (scented water).

### Description of the Experimental Units

#### Device #1

**Figure 1:**
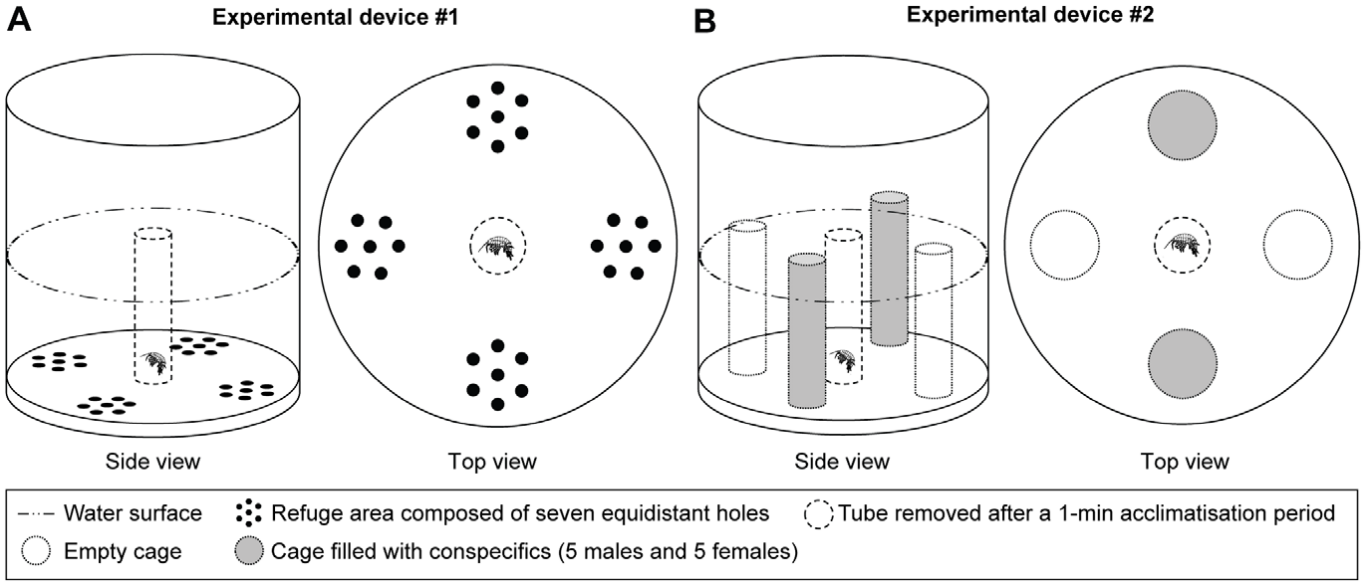
Schematic views of the experimental devices. A) device #1, used to assess hiding, exploration and mobility behaviour; B) device #2, used to assess hiding, aggregation and mobility behaviour.

The first experimental unit comprised a plastic cylindrical tank (25 cm diameter, 31 cm high) that was open to the air and filled with 6 L of test solution (providing a water level of 11 cm; Figure 1A). A round Plexiglas plate (25 cm diameter, 13 mm high), drilled so as to create four equidistant groups of holes 2 cm from the periphery (hereafter called refuge areas), was placed at the bottom of the unit. Each refuge area comprised seven equidistant holes (8 mm diameter, 10 mm deep) inside which amphipods could take refuge by hiding its entire body.

#### Device #2

The second experimental unit used a similar plastic cylindrical tank as for device #1, also filled with 6 L of test solution (Figure 1B). In order to test for aggregative behaviour of amphipods with conspecifics, four equidistant plastic cylindrical cages (4 cm diameter, 13 cm high, 1 mm mesh) were placed vertically on the bottom of the unit, 2 cm from the edge. Two diametrically opposed cages received 10 conspecifics (five individuals of each gender) and the two others were kept empty.

### Behavioural Tests

Each experimental device was placed separately into an hermetic box (50×46×62 cm) fitted with three red-light emitting lamps and a digital video camera set to film the horizontal plan (see [34], [38]). Red light was preferred to white light as amphipods display negative phototaxis. Before starting the test, a translucent plastic tube (3 cm diameter, 14 cm high), open to the air, was placed in the middle of the experimental unit and a single amphipod was introduced into the tube using a fine brush. After a 1-min acclimatisation period, the tube was removed and filming proceeded for 3-min at a rate of 20 frames per second. Each amphipod was used only once and experiments were conducted first with unscented and then with scented water. Each test was repeated 30 times per gender, representing 360 individuals for all experiments. The water was renewed after 5 amphipods had been tested and a 90° rotation of the experimental unit was performed in order to avoid a position effect. During aggregation tests with experimental device #2, the 10 conspecifics enclosed in the cages were not changed throughout the experiment to keep the level of attractiveness constant. At the end of each experiment, all tested amphipods were measured to the nearest mm (from the tip of the rostrum to the base of the telson) under a binocular magnifying glass (Leica MZ 125) fitted with an eyepiece micrometer (Zeiss, 10×). To test the potential effect of gender on the measured responses, amphipod gender was determined *a priori* based on the size of gnathopods (typically larger in males) and was confirmed *a posteriori* by examining the 7th ventral segment (genital papillae only present in males).

In the first experiment, device #1 allowed us to assess (1) hiding, by measuring the proportion of time spent prostrate inside holes; (2) environmental exploration, by counting the number of refuge areas used by a single amphipod; and (3) mobility, by measuring the proportion of time spent moving when outside of refuges. A refuge was considered as used by amphipods only when 80% of its body (excluding antennae) was inside a hole for a period greater than one second.

For the second experiment, we analysed the videos made using device #2 to assess (1) hiding, by first measuring the proportion of time spent close to all cages (i.e. both empty cages and cages filled with conspecifics); (2) aggregation, by focusing on periods during which amphipods were attached to cages and by measuring the proportion of time spent attached to cages containing conspecifics; and (3) mobility, by measuring the proportion of time spent moving when outside of refuges. Individuals were considered as hidden in a refuge area only when immobile and clinging to a cage for a period greater than one second.

### Biomarker Measurement

After each behavioural test, ten pooled samples of 3 males and ten pooled samples of 3 females were prepared in order to analyse energy reserves and MDA levels [40]. Each pooled sample was frozen in liquid nitrogen and stored at –80°C awaiting biomarker measurement. Measurements were performed for females and males separately.

#### Sample preparation

Each pooled sample was homogenised with a manual Potter Elvejhem tissue grinder in 50 mM phosphate buffer KH_2_PO_4_/K_2_HPO_4_ (pH 7.6) supplemented with 1 mM phenylmethyl sulphonylfluoride (PMSF) and 1 mM L-serine-borate mixture as protease inhibitors and 5 mM phenylglyoxal as a γ-glutamyl transpeptidase inhibitor. The homogenisation buffer was adjusted to a volume of two times the wet weight of the pooled sample (i.e. 200 µL of homogenisation buffer for 100 mg of wet weight tissue). The total homogenate was divided into two parts in order to measure each of the different parameters. For each replicate, two independent measures were conducted for each biomarker.

#### Energy reserve assessment

Measurement of total lipids and glycogen content was adapted from [41]. Twenty µL of 2% sodium sulphate (w/v) and 540 µL of chloroform/methanol at 1∶2 (v/v) were added to 40 µL of total homogenate. After 1 h on ice, samples were centrifuged at 3000×*g* for 5 min at 4°C. The resulting supernatant and pellet were used to determinate total lipid and glycogen content, respectively. One hundred µL of supernatant was transferred into a culture tube and placed into a dry bath at 95°C to evaporate the solvent, following which 200 µL of 95% sulphuric acid was added to each tube and left for 10 min. The culture tubes were then cooled on ice before addition of 4.8 mL of a vanillin-phosphoric acid reagent. After 10 min of reaction, absorbance was measured at 535 nm. Commercial cholesterol was used as a standard and lipid content was expressed in mg/mL. Total dissolution of the pellet was performed in 400 µL of deionized water for 10 min in an ultrasonic bath. One hundred µL of sample was placed into culture tubes and 4.9 mL of anthrone reagent added. The mixture was placed into a dry bath at 95°C for 17 min and then cooled on ice. Absorbance was measured at 625 nm, with glucose used as a standard and concentrations expressed in µg/mg wet weight.

#### Toxic effect biomarker

MDA levels were measured using a high-pressure liquid chromatography (HPLC) method adapted from [42], with UV detection set at 267 nm. Seventy µL of the total homogenate was deproteinised by diluting four-fold with 95% ethanol (HPLC grade) and cooling on ice for 1.5 h. The mixture was then centrifuged at 18,000×*g* for 30 min at 4°C. One hundred µL of the resultant supernatant was injected directly into a reversed-phase LiChrospher 100RP18-encapped HPLC column. Separation was conducted at 25°C and elution was carried out with sodium phosphate buffer (pH 6.5) containing 25% ethanol and 0.5 mM tetradecylmethylammoniun bromide as an ion-paring reagent. MDA levels were expressed in ng MDA/mg lipid.

### Data Analysis and Statistics

As all data met assumptions of normality and homogeneity of variance, each response was analysed using ANOVA, followed by the post-hoc Tukey HSD test. All tests were performed with a 5% type I error risk, using R 2.9.0 Software. To verify if significant relationships existed between behavioural indices and individual lengths, correlations were performed and tested using Pearson correlation tests (not illustrated). As no significant differences were observed between males and females during behavioural tests, we decided to pool the genders for each condition in order to make the results more robust.

## Results

### Toxic Effect Biomarker

**Figure 2:**
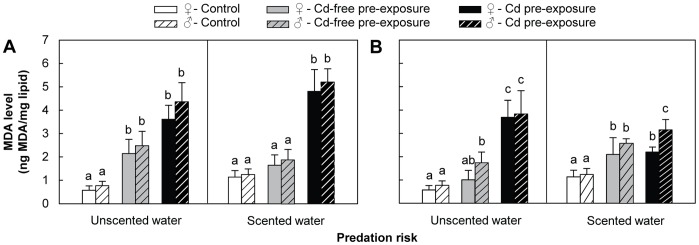
Malondialdehyde (MDA) levels measured in the presence/absence of predation risk. A) device #1; B) device #2. *D. villosus* females and males are represented by filled and hatched bars, respectively. MDA levels were measured following behavioural tests in which amphipods were exposed to the following conditions: Control = 24-h pre-exposure to Cd-free water +4-min test exposure to Cd-free water (white bars); Cd-free pre-exposure = 24-h pre-exposure to Cd-free water +4-min test exposure to 500 µg Cd/L (grey bars); Cd pre-exposure = 24-h pre-exposure to 500 µg Cd/L +4-min test exposure to 500 µg Cd/L (black bars). All data are means of *N* = 10± SD. Significant differences are indicated by different letters (Tukey HSD post-hoc test after ANOVA, *P*<0.05).

The results of three-factor ANOVA analysis are presented in Table 1. In device #1, Tukey HSD post-hoc tests indicated that, without predation risk, males and females from the Cd-free pre-exposure and Cd pre-exposure groups had more than twofold higher MDA levels than control gammarids (Figure 2A). No significant differences were observed between Cd-free pre-exposure and Cd pre-exposure groups. In the presence of predation risk, MDA levels in both genders of the Cd pre-exposure group showed a significant increase of more than threefold compared to the control. No significant differences were observed between the control group and the Cd-free pre-exposure group. In addition, no significant inter-sex differences were observed, whatever the pre-exposure/exposure conditions.

**Table 1:** Analysis of variance (three-factor ANOVA) in biomarkers for toxicity (MDA) and energetic reserves (lipid and glycogen) in *D. villosus*, according to gender, predation risk and cadmium (Cd) contamination.

Experimental unit	Parameter	Source of variation	DF	*F*	*P*
Device #1	MDA	Gender	1	4.57	**0.0349**
		Predation risk	1	13.88	**<0.001**
		Cd	2	223.16	**<0.001**
		Gender × Predation risk	1	0.002	0.9604
		Gender × Cd	2	0.40	0.6701
		Predation risk × Cd	2	161.15	**<0.001**
		Gender × Predation risk × Cd	2	0.06	0.9377
	Lipid	Gender	1	101.20	**<0.001**
		Predation risk	1	471.87	**<0.001**
		Cd	2	66.95	**<0.001**
		Gender × Predation risk	1	3.52	0.0635
		Gender × Cd	2	1.96	0.1462
		Predation risk × Cd	2	104.88	**<0.001**
		Gender × Predation risk × Cd	2	4.33	**0.0155**
	Glycogen	Gender	1	193.78	**<0.001**
		Predation risk	1	168.29	**<0.001**
		Cd	2	40.06	**<0.001**
		Gender × Predation risk	1	1.92	0.1688
		Gender × Cd	2	1.09	0.3402
		Predation risk × Cd	2	82.68	**<0.001**
		Gender × Predation risk × Cd	2	9.13	**<0.001**
Device #2	MDA	Gender	1	52.05	**<0.001**
		Predation risk	1	2.25	0.1364
		Cd	2	276.66	**<0.001**
		Gender × Predation risk	1	0.32	0.5715
		Gender × Cd	2	7.65	**<0.001**
		Predation risk × Cd	2	69.47	**<0.001**
		Gender × Predation risk × Cd	2	0.64	0.5273
	Lipid	Gender	1	106.75	**<0.001**
		Predation risk	1	338.02	**<0.001**
		Cd	2	82.21	**<0.001**
		Gender × Predation risk	1	8.11	**0.0052**
		Gender × Cd	2	1.76	0.1774
		Predation risk × Cd	2	64.05	**<0.001**
		Gender × Predation risk × Cd	2	8.03	**0.0006**
	Glycogen	Gender	1	205.04	**<0.001**
		Predation risk	1	93.58	**<0.001**
		Cd	2	95.04	**<0.001**
		Gender × Predation risk	1	7.59	**0.0069**
		Gender × Cd	2	14.37	**<0.001**
		Predation risk × Cd	2	72.01	**<0.001**
		Gender × Predation risk × Cd	2	3.54	**0.0323**

Significant values are shown in bold.

In device #2, Tukey HSD post-hoc tests indicated that, without predation risk, MDA levels were significantly increased by twofold in males of the Cd-free pre-exposure group and more than fivefold in both genders from the Cd pre-exposure group, when compared to the control (Figure 2B). Gammarids pre-exposed to Cd showed significantly higher MDA levels than those not pre-exposed. In scented water, MDA levels of both genders in the Cd-free pre-exposure and Cd pre-exposure groups increased significantly by more than twofold compared to the control. A significant difference between the Cd-free pre-exposure and Cd pre-exposure groups was only observed in males, however, with MDA levels being higher in the Cd pre-exposure group. In scented water, a significant inter-sex difference was only observed in the Cd pre-exposure group, with MDA levels being significantly higher in males.

### Behavioural Responses

**Figure 3:**
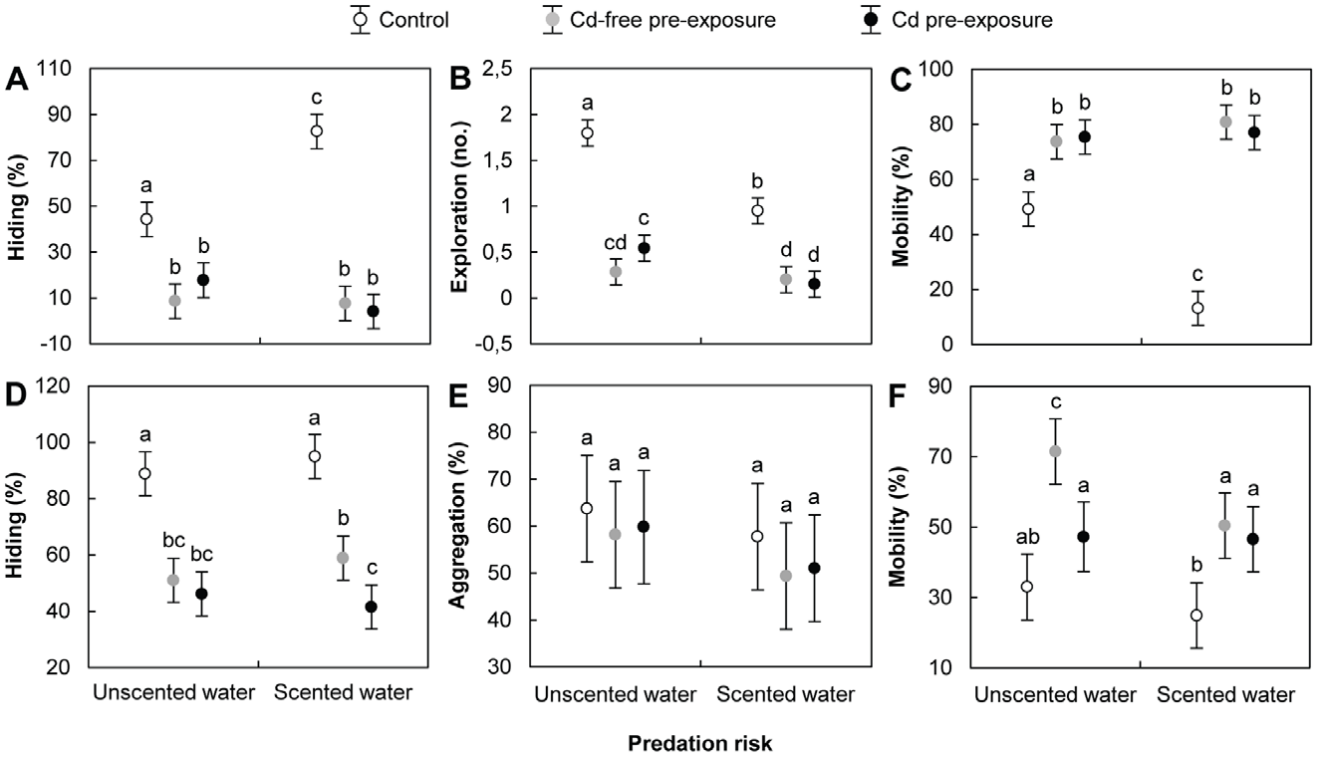
Behavioural responses measured in the presence/absence of predation risk. A) hiding in device #1 (i.e. proportion of time spent in holes); B) environmental exploration in device #1 (i.e. number of refuge areas used); C) mobility in device #1 (i.e. proportion of time spent moving when outside of refuges); D) hiding in device #2 (i.e. proportion of the time spent clinging to cages); E) aggregation in device #2 (i.e. proportion of the time spent clinging to cages filled with conspecifics); F) mobility in device #2 (i.e. proportion of the time spent moving when outside of refuges). *D. villosus* were exposed to the following conditions: Control = 24-h pre-exposure to Cd-free water +4-min test exposure to Cd-free water (white dots); Cd-free pre-exposure = 24-h pre-exposure to control water +4-min test exposure to 500 µg Cd/L (grey dots); Cd pre-exposure = 24-h pre-exposure to 500 µg Cd/L +4-min test exposure to 500 µg Cd/L (black dots). All data are means of *N* = 60±95% confidence intervals. Significant differences are indicated by different letters (Tukey HSD post-hoc test after ANOVA, *P*<0.05).

The results of two-factor ANOVA analysis are presented in Table 2. In device #1, Tukey HSD post-hoc tests indicated that, whether in unscented or scented water, hiding and exploration decreased significantly by more than threefold, while mobility increased significantly by more than 50%, in Cd-free pre-exposure groups compared to the control (Figures 3A, B, C). For the Cd pre-exposure groups, hiding and exploration decreased significantly by more than twofold and mobility increased significantly by more than 50% compared with the control. No significant difference was observed in hiding, exploration and mobility values between the Cd-free pre-exposure and Cd pre-exposure groups. In the control groups, values for exploration and mobility decreased significantly by more than twofold, while hiding increased significantly by twofold, in scented water. No significant effect of predation risk was observed on antipredator behaviour in both the Cd-free pre-exposure and Cd pre-exposure groups, except that exploration decreased significantly by threefold in the Cd pre-exposure groups in scented water.

**Table 2:** Analysis of variance (two-factor ANOVA) in hiding, exploration and mobility behaviour in *D. villosus* measured with device #1, and hiding, aggregation and mobility behaviour measured with device #2, in response to predation risk and cadmium (Cd) contamination.

Experimental unit	Parameter	Source of variation	DF	*F*	*P*
Device #1	Hiding	Predation risk	1	8.22	**0.0095**
		Cd	2	63.57	**<0.001**
		Predation risk × Cd	2	1.74	0.1770
	Exploration	Predation risk	1	57.92	**<0.001**
		Cd	2	62.29	**<0.001**
		Predation risk × Cd	2	15.05	**<0.001**
	Mobility	Predation risk	1	65.48	**<0.001**
		Cd	2	70.74	**<0.001**
		Predation risk × Cd	2	8.13	**0.0156**
Device #2	Hiding	Predation risk	1	0.92	0.3382
		Cd	2	79.90	**<0.001**
		Predation risk × Cd	2	1.45	0.2367
	Aggregation	Predation risk	1	2.71	0.1007
		Cd	2	0.80	0.4519
		Predation risk × Cd	2	0.04	0.9603
	Mobility	Predation risk	1	6.48	**0.0113**
		Cd	2	22.98	**<0.001**
		Predation risk × Cd	2	2.31	0.1006

Significant values are shown in bold.

In device #2, Tukey HSD post-hoc tests indicated that, whether or not water was scented, hiding in the Cd-free pre-exposure groups decreased significantly by around 40%, while mobility values increased significantly by more than twofold, compared with the controls (Figures 3D, F). In the Cd pre-exposure groups, hiding values decreased significantly by more than two fold and mobility values increased significantly by more than 40% compared to the control. Significant differences between the Cd-free pre-exposure and Cd pre-exposure groups were only observed for hiding in scented water and for mobility in unscented water. In both cases, values in the Cd pre-exposure groups were lower than in the Cd-free pre-exposure groups. No significant differences were observed in aggregation, whatever the test conditions (Figure 3E). No significant effect of predation risk was observed on antipredator behaviour in the three groups, except that, in the Cd-free pre-exposure groups, mobility was significantly lower in scented water.

In device #1, length ranges for *D. villosus*, respectively with and without predation risk, were 15.32–19.08 and 14.66–20.00 mm in the control group; 15.00–19.50 and 16.88–20.39 mm in the Cd-free pre-exposure group; and 16.38–19.63 and 14.75–19.00 mm in the Cd pre-exposure group. In device #2, length ranges of *D. villosus*, respectively with and without predation risk, were 15.32–19.08 and 15.97–19.21 mm in the control group; 15.96–20.76 and 14.63–20.63 mm in the Cd-free pre-exposure group; and 16.64–19.50 and 16.47–21.06 mm in the Cd pre-exposure group. No significant effect of gammarid length was observed for hiding, exploration or mobility in device #1, and for hiding, aggregation or mobility in device #2 (Pearson correlation tests, *P*>0.05; not illustrated).

### Energy Reserves

**Figure 4:**
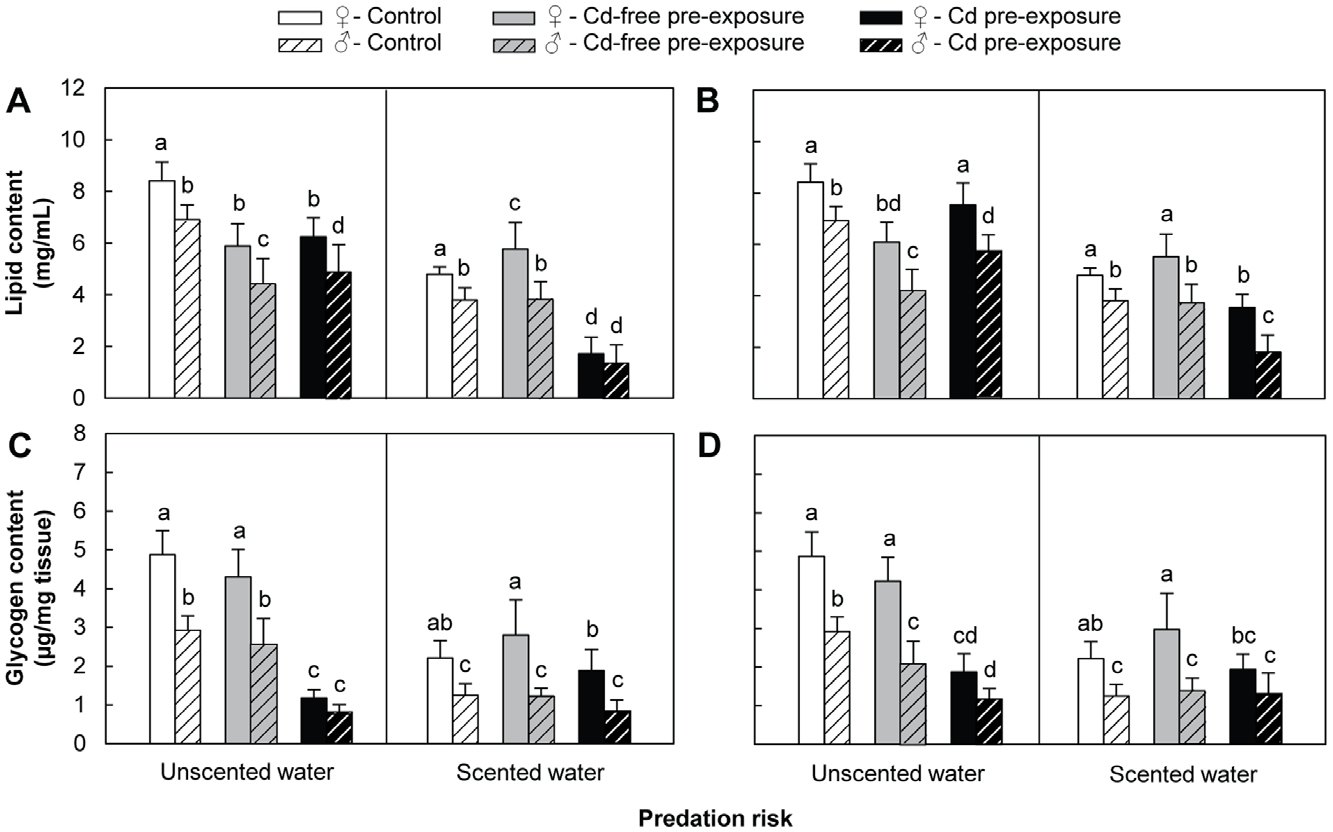
Energy reserves measured in the presence/absence of predation risk. A) lipid content in amphipods from device #1; B) lipid content in amphipods from device #2; C) glycogen content in amphipods from device #1; D) glycogen content in amphipods from device #2. *D. villosus* females and males are represented by filled and hatched bars, respectively. Energy reserves were measured following behavioural tests in which amphipods were exposed to the following conditions: Control = 24-h pre-exposure to Cd-free water +4-min test exposure to Cd-free water (white bars); Cd-free pre-exposure = 24-h pre-exposure to Cd-free water +4-min test exposure to 500 µg Cd/L (grey bars); Cd pre-exposure = 24-h pre-exposure to 500 µg Cd/L +4-min test exposure to 500 µg Cd/L (black bars). All data are means of *N* = 10± SD. Significant differences are indicated by different letters (Tukey HSD post-hoc test after ANOVA, *P*<0.05).

Results for three-factor ANOVA analysis are presented in Table 1. In device #1, Tukey HSD post-hoc tests revealed that, without predation risk, lipid content in both the Cd-free pre-exposure and Cd pre-exposure groups decreased significantly by about 30% compared to the control (both genders). No significant differences were observed between the Cd-free pre-exposure and Cd pre-exposure groups (Figure 4A).

Values for both genders in the Cd pre-exposure group showed a significant threefold decrease in glycogen content compared to both the control and the Cd-free pre-exposure group. No significant differences were observed between the control and the Cd-free pre-exposure group (Figure 4B). In the presence of predation risk, lipid content was significantly higher only in females of the Cd-free pre-exposure group and decreased significantly by threefold for both genders in the Cd pre-exposure group, compared to the control. For both genders, glycogen content did not differ significantly from the control in both the Cd-free pre-exposure and Cd pre-exposure groups. Values for females in the Cd pre-exposure group, however, were significantly lower than those in the Cd-free pre-exposure group. In both scented and unscented water, significant inter-sex differences were observed for all responses (female energy reserves being higher than those of males), except for lipid content in the Cd pre-exposure group in scented water and for glycogen content in the Cd pre-exposure group for unscented water.

In device #2, Tukey HSD post-hoc tests revealed that, without predation risk, lipid content values for both genders decreased significantly by more than 25% in the Cd-free pre-exposure group, while in the Cd pre-exposure group, lipid content decreased by around 10% in males only compared to the control. A significant difference was observed in both genders between the Cd-free pre-exposure and Cd pre-exposure groups, with lipid content in the Cd pre-exposure group being higher (Figure 4C).

Glycogen content values decreased significantly by around 30% in males in the Cd-free pre-exposure group and decreased significantly by more than twofold for both genders in the Cd pre-exposure group, compared to the control. Individuals in the Cd pre-exposure group contained significantly less glycogen than those in the Cd-free pre-exposure group (Figure 4D). In scented water, lipid content decreased significantly by more than 20% in both genders in the Cd pre-exposure group, compared with both the control and the Cd-free pre-exposure group. No significant differences were observed, however, between the control group and the Cd-free pre-exposure group. Glycogen content in both the Cd-free pre-exposure and Cd pre-exposure groups did not differ significantly from the control in both genders, though values for females from the Cd pre-exposure group were significantly lower than those of the Cd-free pre-exposure group. Whether scented or unscented water, significant inter-sex differences were found for all responses (female energy reserves being higher than those of males), except for glycogen content in the Cd pre-exposure group for both scented and unscented waters.

## Discussion

### Cadmium Responses

#### Toxic effect biomarker

Cd clearly had a significant effect on MDA values, with levels in *D. villosus* increasing upon exposure to the heavy metal in both experimental devices. A significant rise in MDA level has previously been reported for the amphipod *Gammarus pulex* after exposure to 300 µg Cd/L for 13 days [43]; and also in bivalves such as *Ruditapes decussatus* following exposure to 4, 40 and 100 g Cd/L for 28 days [44] and both *Crassostrea gigas* and *Mytilus edulis* exposed to 200 µg Cd/L for 21 days [45]. MDA content is a reliable indicator of cell damage as it is a final product of membrane lipid degradation (i.e. lipoperoxidation). Indeed, the higher the MDA level the higher the level of cell damage [46]. Cd is known to generate reactive oxygen species that may act as signalling molecules in the induction of gene expression and apoptosis [47] and deplete endogenous radical scavengers. Cd is also known to cause damage to a variety of transport proteins including Na^+^/K^+^-ATPase, as has been demonstrated for *G. pulex* [48]. Our results indicated no significant difference between males and females, suggesting that responses to stress may not be gender-dependant in *D. villosus*, contrary to what has been shown in other amphipod species exposed to various stressors [34], [35]. In addition, our study highlighted the rapidity of adverse effects to Cd, with significant increases in MDA levels being measured directly after a 4-min test exposure to 500 µg Cd/L without Cd pre-exposure.

#### Behavioural responses

Our results revealed a significant increase in *D. villosus* locomotion in both experimental devices when exposed to Cd, with both refuge use and exploratory activity decreasing, and mobility increasing when outside of refuges. Such stress-induced responses can be interpreted as a rapid evasion tactic and, in the field, may represent the first line of defence that reduces the probability of contact with the toxicant (e.g. upstream movement). In contrast, a significant decrease in locomotor activity has been reported in *G. pulex* following 120-h exposure to 7.5 µg Cd/L [48], and in *G. lawrencianus* following 72-h exposure to 62 µg Cd/L [49], Cd concentrations that are ten- to fiftyfold lower than those used in this study. Differences to previous studies may be attributed to the assessment of a quasi-immediate/short-term locomotor response to Cd ecotoxicity in our experiments, which may reflect behaviour expressed under sudden discharges of highly concentrated toxic elements under natural conditions. Moreover, a 24-h pre-exposure to Cd did not result in any significant enhancement in behavioural response relative to that induced following a 4-min test exposure. Acclimation to Cd may explain why 24-h pre-exposed individuals were as mobile as instantaneously-exposed individuals. Indeed, a resistance to acute Cd ecotoxicity has been demonstrated in *G. pulex* pre-exposed for 24 h to different concentrations [50]. The induction of metallothionein-like proteins, which can sequester Cd in a less toxic form, is one of the most plausible hypotheses to explain the physiological acclimation of gammarids to Cd [51].

#### Energy reserves

In our study, total lipid and glycogen contents were assessed to estimate energy reserves of individuals. Lipids are stored in fat bodies and used during starvation and/or reproduction periods [52] while glycogen generally provides energy resources for short-term activities [53]. Both can be easily catabolised to supply the energy necessary for antitoxic defences involved in defence mechanisms [54]. Our results revealed that Cd affected energy reserves, especially when *D. villosus* individuals were pre-exposed. A similar decrease in both lipid and glycogen content has also been highlighted in *G. roeseli* exposed to Cd [40], and a decrease in lipid content has been observed in in *Daphnia magna* following a 48-h exposure to Cd [55]. The decrease in energy content in organisms exposed to Cd could be explained by the energetic cost of tolerance offsetting the stress produced by the toxicant [54], [56] as lipids and glycogen are both known to be first mobilised and rapidly used by organisms exposed to stressors [57]. A cumulative hypothesis might be the use of lipid and glycogen as an energy source for the increased locomotor activity observed in gammarids exposed to Cd. Our results also revealed higher energy reserves in females compared to males. These gender-based energy reserves differences can be explained by the reproductive process, oogenesis in females being more costly than spermatogenesis in males.

### Responses to Predation Risk

Our results indicate that, in the absence of Cd, *D. villosus* were able to sense the alarm stimuli as, when exposed to chemical cues from a fish predator and/or injured conspecifics, individuals expressed a more cautious behavioural strategy by (1) increasing the time spent hiding in holes, (2) decreasing exploratory activity (i.e. visiting refuge areas less), and (3) decreasing mobility when outside of refuges. These responses are in accordance with a general decrease in locomotor activity and drift rate reported for several gammarid species exposed to predation risk [39], [58], [59]. Minimising displacement is believed to reduce the risk of being spotted by a potential predator, and especially by a fish predator. As hypothesised in [60], fish predators may be avoided by hiding in dense structures and/or by decreasing activity levels, as fish swim faster than amphipods. The absence of aggregative behaviour in *D. villosus*, an efficient means reducing individual risk of predation, is coherent with the species’ aggressiveness and voracity towards potential prey, including conspecifics, reported by other authors [61], [62].

In Cd spiked water, gammarids exposed to predation risk spent most time outside of refuges and were more mobile when outside of refuges. The absence of antipredator behavioural adjustments following Cd exposure could be explained by a disruption in the transfer of chemical information. The deregulation of internal information transfer signalling pathways by chemical pollutants is well known in various organisms across the taxonomic spectrum [63] and concerns not only chemical information within animals (i.e. endocrine disruption) but also chemical information transfer between organisms. Several studies have demonstrated disruption of chemically induced antipredator responses by pollutants such as pesticides [64], surfactants [63] and heavy metals, which have all been shown to impair predator avoidance behaviour in fish [65], aquatic snails [66], amphibians [67] and water fleas [68]. In fish, for example, Cd has been demonstrated to significantly affect (1) environmental exploration performance and behavioural reactions to conspecific chemical communication in *Brycon amazonicus* exposed for 96 h to 9.04±0.07 µg Cd/L [6], and (2) the ability of *Oncorhynchus mykiss* to respond to natural pheromones after being exposed for 7 days to 2 µg Cd/L [65]. Even if the disruption mechanisms remain unresolved, three hypotheses can be proposed here: (1) a direct alteration to kairomone chemistry by Cd, (2) out-competing of kairomone molecules by Cd at the receptor sites, or (3) interference by Cd with some process along the signal transduction pathway from kairomone reception to behavioural response. Such impairments to chemosensory functioning may have a strong effect on highly ecological functions. Organisms use chemical messengers not only to detect enemies but also to sense prey and to locate food, obtain mates, recognise close kin or mark a territory and impairment could lead to important ecological perturbations in populations inhabiting contaminated systems. We would suggest that assessing whether the absence of antipredator behavioural responses in Cd-exposed gammarids significantly compromises their survival when facing a predator should be the next step investigated. Additionally, more research is needed to determine the large-scale ecological implications of chemosensory dysfunction in metal-contaminated systems.

### Conclusions

The results of this study demonstrate that 24-h exposure of *D. villosus* to Cd at non-lethal concentrations can (1) generate cell damage as revealed by higher MDA levels in exposed individuals, (2) induce short-term behavioural changes in locomotion, and (3) affect energy reserves. Our results also highlight that short-term Cd exposure can alter the olfactory-mediated behavioural responses of a freshwater gammarid to alarm substances from a predator or conspecifics. As a result, Cd could have important implications for predator avoidance strategies and, quite possibly, could affect other aspects of the animal–environment relationship, e.g. *D. villosus* prey population success, the leaf litter breakdown process, or toxicant biomagnification along the food chain. The mechanisms involved in Cd disturbance of olfactory functioning, depletion of energy reserves, and degradation of membrane lipid should be the subject of further studies.
